# Reconstruction of Sphenoid Wing Dysplasia in Neurofibromatosis Type-1 Patients: An Evolving Technique

**DOI:** 10.1016/j.jpra.2021.10.002

**Published:** 2021-11-10

**Authors:** Mr Naveen Virin Goddard, Mr Jonathan Dunne, Mr Samim Ghorbanian, Mr Simon Eccles

**Affiliations:** a University of Birmingham, Birmingham, B15 2TT; b Chelsea and Westminster Hospital, 369 Fulham Road, London, SW10 9NH

**Keywords:** Neurofibromatosis type-1, sphenoid wing dysplasia, reconstruction, titanium implant, arachnoid cyst decompression

## Introduction

Neurofibromatosis type-1 (NF-1) is a genetic disorder affecting 1 in 2500 to 1 in 3000 people worldwide.^1^ NF-1 causes dysplasia of bone in addition to soft tissues, and sphenoid wing dysplasia is one such manifestation, occurring in 3–11% of patients.^2^ It is characterised by changes in the orbital volume, and progressive proptosis secondary to herniation of the temporal lobe into the orbit; however, reports of surgical correction are sparse. Sequelae are devastating, with abnormal ocular movements, optic nerve stretch, and ultimately loss of vision.

The aim of this study was to report the evolution of a novel reconstructive technique for sphenoid wing dysplasia in four patients with NF-1.

## Materials and Methods

All patients with NF-1 presenting to a single national referral unit between 1st January 2015 and 31st December 2020 were included. Data were reviewed retrospectively, and four patients who underwent surgical correction of sphenoid wing dysplasia were identified. A database was established to record patient demographics, surgical technique, complications, and visual and aesthetic outcomes.

## Surgical Technique

**Figure 1:**
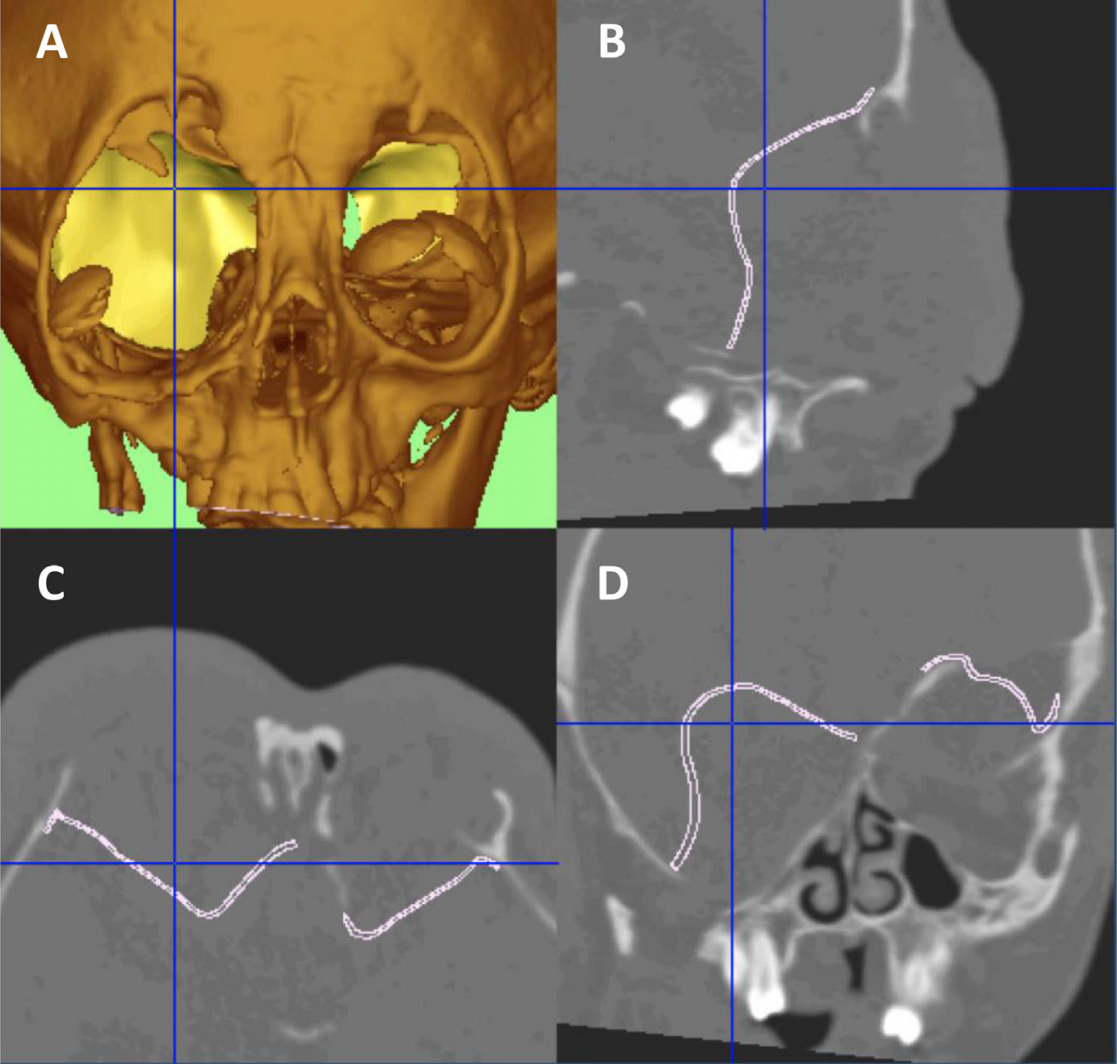
**(A)** 3D CT reconstruction of the sphenoid wing dysplasia defect. **(B, C, D)** CT planning of the custom-made titanium implants.

A full pre-operative assessment of the patient is undertaken. Fine cut head and face computed tomography (CT) scans with contrast are performed to delineate the defect (Figure 1 A–D), and a custom-made three-dimensional (3D) printed titanium implant is developed based on the contralateral sphenoid bone in unilateral disease. In bilateral disease, it is modelled to reconstruct the defect as a best fit.

The patient is positioned supine and craniofacial infiltration injected within maximal dose limits. A bicoronal incision is used to elevate the following: (1) an anteriorly based sub-Galeal flap to the junction of the superior orbital rim and orbital roof and (2) an anteriorly based pericranial flap. Temporo-parietal craniotomy is performed and the dura mater is incised. The associated arachnoid cyst of the temporal lobe is decompressed endoscopically to reduce the cerebral volume herniating through the dysplastic sphenoid wing, facilitating exposure of the defect. The titanium implant is secured to the margins of the defect with unicortical screws (Supplementary Figure 1 A, B), and the craniotomy bone flap is secured with titanium plates. A subcutaneous redivac drain is inserted, and the skin is closed with 4/0 Vicryl deep dermal sutures, with 4/0 Vicryl Rapide in children and staples in adults. A head bandage is applied for 48 h. The drain is removed at 48 h, or until drainage is less than 30 ml per 24 h.

## Results

Four patients had sphenoid wing dysplasia requiring orbital reconstruction, with one patient having bilateral sphenoid wing reconstruction (Supplementary Figure 2). Two patients had no vision preoperatively, one had unchanged limited vision postoperatively, one had resolution of epiphora postoperatively and one had unchanged vision with subsequent surgery for a pre-existing left convergent squint.

The first two cases had craniotomy and reconstruction with a non-anatomical titanium mesh. Access was difficult in these cases, and defining the posterolateral border of the defect required two assistants, with a less anatomically precise reconstruction, differing to the non-pathological side. The final three cases, performed on two patients, had endoscopic decompression of the arachnoid cyst to improve exposure of the defect, and a custom-made titanium 3D printed implant. Access and implant placement was significantly improved, and reconstruction modelled on the contralateral side, or a computer-based reconstruction in the bilateral case. One patient had a cerebrospinal fluid (CSF) leak, successfully managed with a shunt. Pre- and postoperative clinical photographs of two patients are shown in Supplementary Figure 3 (Supplementary Figure 3 A–I) and Supplementary Figure 4 (Supplementary Figure 4 A–J).

## Discussion

This series details our management of sphenoid wing dysplasia in patients with NF-1 and is one of the largest reported to date. Orbital reconstruction in sphenoid wing dysplasia is a sight-preserving procedure, reducing optic nerve stretch, correcting proptosis and buphthalmos, and improving appearance. Our evolution towards arachnoid cyst decompression has significantly improved access and reduced the volume of temporal lobe prolapse. In addition, our refined reconstructive approach has accurately restored orbital volume using custom-made titanium implants.

At present, there is still no accepted gold standard for the surgical management of the defects resulting from sphenoid wing dysplasia.

In our centre, all patients are managed by a multidisciplinary team, with every relevant medical, surgical and psychological subspecialties involved throughout. In our practice, surgical intervention is guided by visual symptoms, rate of expansion of orbital volume and degree of proptosis. Evidence of progressive visual impairment in conjunction with temporal lobe herniation due to sphenoid wing dysplasia mandates surgery (Supplementary Figure 5 A–D). This cohort of patients presented to us with sphenoid wing dysplasia and established herniation with visual deterioration, and therefore surgery was required. Despite two patients having no vision preoperatively, surgery was still indicated to arrest the advancing herniation (Supplementary Figure 6 A–I). Where sphenoid wing dysplasia is in its early stages with the absence of visual deterioration, serial imaging is utilised to monitor for change.

The objectives of surgery are to restore the barrier between the orbit and the temporal lobe, thus allowing the preservation of vision and improvement of ocular movement. For many years, titanium mesh has been used to repair defects as it offered a solution to bone resorption and recurrence of symptoms associated with traditional split bone grafts.^3^ However, the use of titanium mesh has been associated with the development of infection, adhesions and recurrence of dural herniation.^4^ Therefore, new techniques have been developed including the use of titanium mesh covered by lyophilised dura, which is reported to prevent the adherence of the mesh and the passage of meningoencephalocele through its permeations.^5^ In our series, we have not had any implant-related infections, and we believe that in the presence of healthy vascularised soft tissue cover, the risk is small.

Other alloplastic materials such as high-density porous polyethylene (HDPP)^4^ and synthetic bone cement (polymethyl methacrylate (PMMA))^6^ have been used as alternatives to titanium mesh. However, both materials have limitations in their ability to contour to the native sphenoid wing and precisely reconstruct the defect.

Use of custom-made 3D printed titanium implants precisely reconstructs the defect and eliminates the need for remodelling intraoperatively, while providing a thin and durable barrier between the cranium and orbit. Furthermore, improved access to the defect created through endoscopic decompression of arachnoid cysts, makes the placement and fixation of the implant simpler.

Restoring orbital volume is a key factor in re-establishing optimal function and cosmesis.^7^ Measurement of orbital volume would be useful to monitor the progression of sphenoid wing dysplasia and provide assessment of pre- and postoperative changes. However, there is still no accepted gold standard for measuring the orbital volume, and therefore, it has not been adopted into our practice.^8^

Targeted medical strategies have the potential to dramatically alter the treatment landscape in NF-1 by reducing the volume of disease prior to surgery or eliminating the need for surgery entirely. Recent results from trials initiated by the Neurofibromatosis Clinical Trials Consortium (NFCTC) have been extremely promising, demonstrating that Selumetinib (KOSELUGO, AstraZeneca, Cambridge, UK), a MEK inhibitor, can significantly reduce the size of NF1-associated plexiform neurofibromas.^9^ Indeed, in a recent phase II clinical trial, Selumetinib had a 72% response rate for volume reduction in inoperable plexiform neurofibromas.^10^

A future in which effective medical treatments for NF-1 tumours are available, alongside surgical interventions, may represent the future of management. However, as the advent of medical therapies to treat NF-1 is in its infancy, surgery remains the mainstay of managing this complex and debilitating condition. The orbital sequelae can be devastating, and our novel technique to reconstruct sphenoid wing dysplasia has durable results with a low complication rate.

Supplementary Figure 1. (A, B) 3D CT reconstructions showing the titanium implants *in situ*.

## Declaration of Competing Interest

None declared

## Appendix Supplementary materials

Supplementary Figure 2. Demographics and surgical outcomes of patients who received sphenoid wing dysplasia reconstruction. Image, application 1

Supplementary Figure 3. (A, B, C) A 13-year-old female that presented with bilateral sphenoid wing dysplasia and herniation into both orbits. (D, E, F) Clinical photographs of the same patient 10 months later, highlighting the rapid advancing herniation into the right orbit. (G, H, I) Clinical photographs taken after bilateral sphenoid wing dysplasia repair using endoscopic decompression and a custom-made 3D titanium implant. Image, application 2

Supplementary Figure 4. (A, B, C, D, E) A 23-year-old male who presented with right sphenoid wing dysplasia with herniation into the right orbit. (F, G, H, I, J) Clinical photographs taken one year after right sphenoid wing repair with titanium mesh. Image, application 3

Supplementary Figure 5. (A) Pre-operative 3D CT reconstruction demonstrating the radiological indications for surgery in one patient. (B, C, D) CT imaging demonstrating the radiological indications for surgery in one patient. Image, application 4

Supplementary Figure 6. (A, B, C) A 2-year-old male patient that presented with left sphenoid wing dysplasia with herniation into the left orbit. Despite absent vision in the left eye at the time of surgery, surgical intervention was still indicated to arrest the progressive herniation demonstrated in consecutive pre-operative photographs of the patient aged 3 (D, E, F) and 5 years old (G, H, I).. Image, application 5

Image, application 6
