# Supplementary figures and images for: Reconstruction of Sphenoid Wing Dysplasia in Neurofibromatosis Type-1 Patients: An Evolving Technique
Source: JPRAS Open. 2021 Nov 10;31:67–71. doi: 10.1016/j.jpra.2021.10.002 (PMC8666329; doi:10.1016/j.jpra.2021.10.002)

**Supplementary Figure 1. (A, B)**


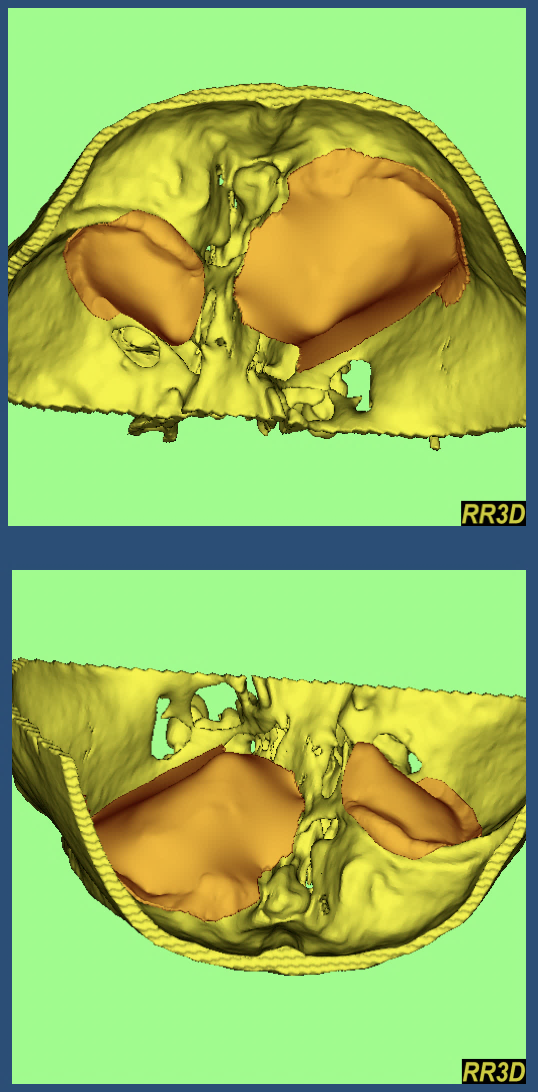

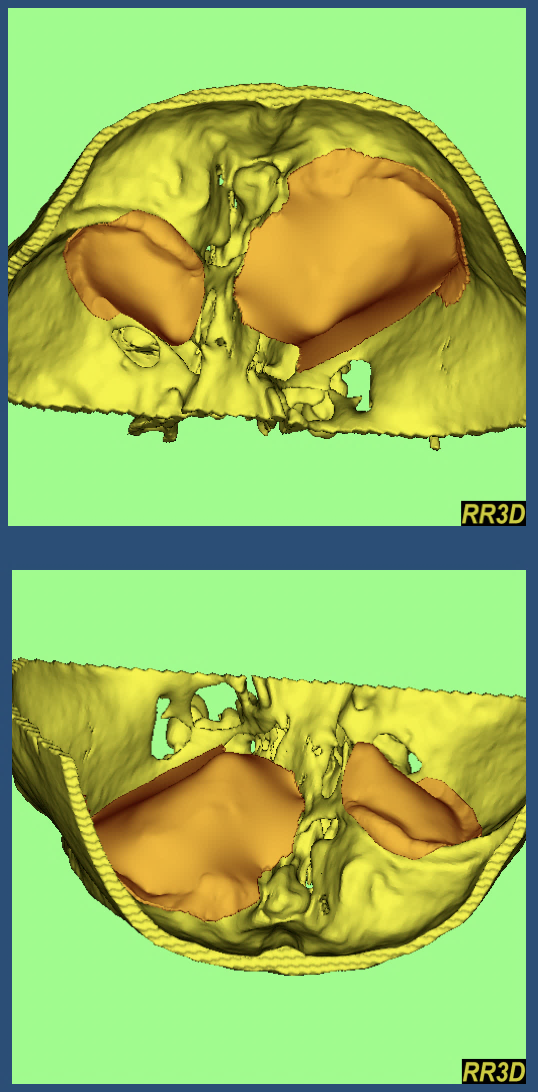


**A**

**B**

Supplement: Supplementary file 1 — Supplementary Figure 2. Demographics and surgical outcomes of patients who received sphenoid wing dysplasia reconstruction. [file mmc1.docx]

**Supplementary Figure 3. (A, B, C, D, E, F, G, H, I)**

**AA**

**IA**

**DA**

**EA**

**FA**

**CA**

**BA**

**HA**

**GA**


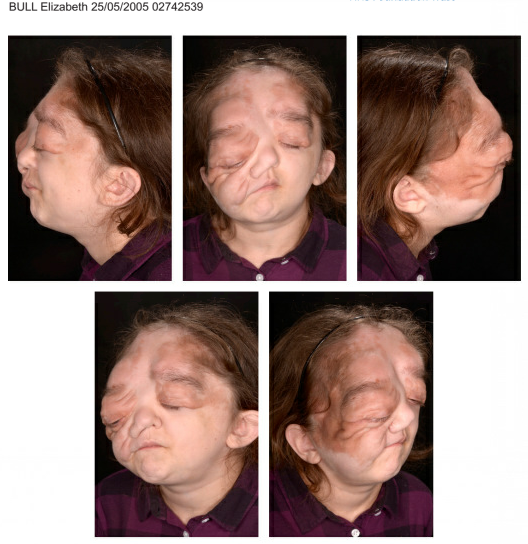

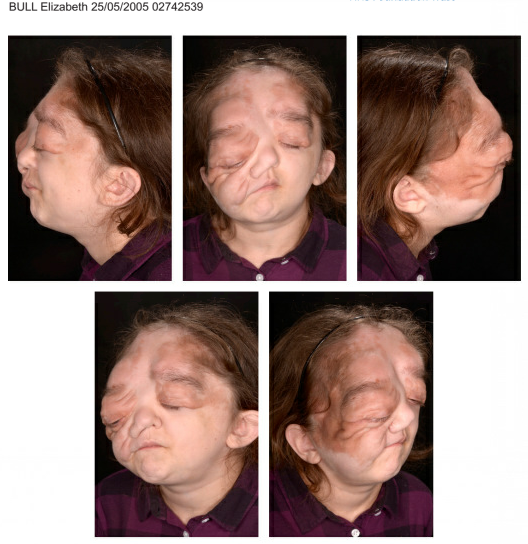

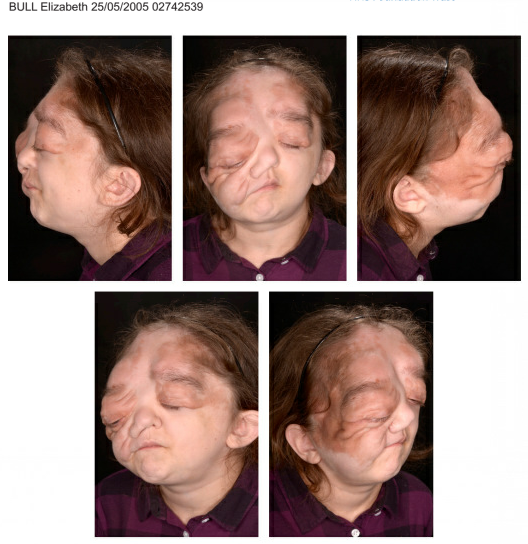

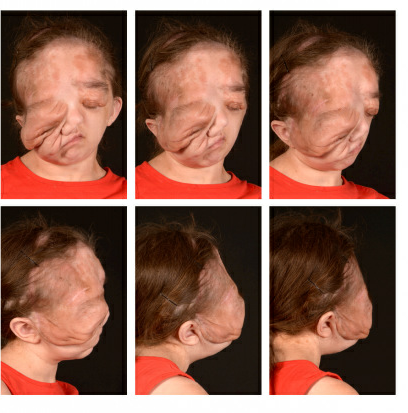

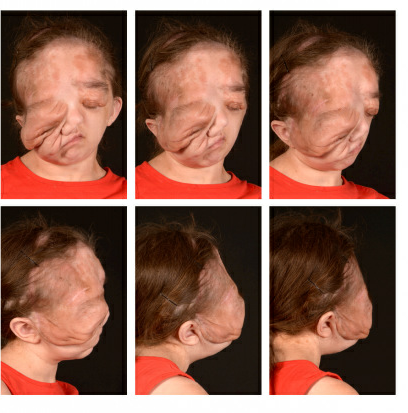

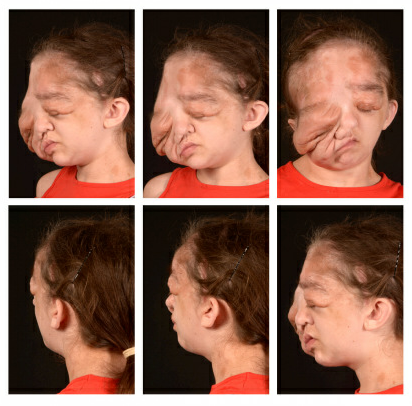

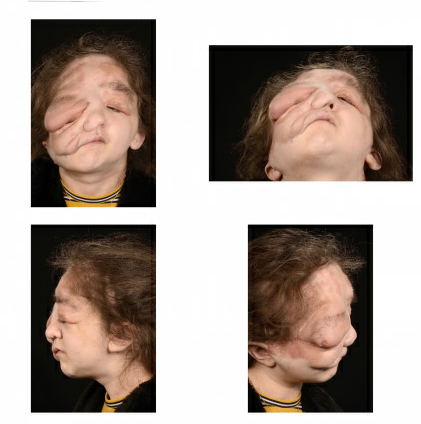

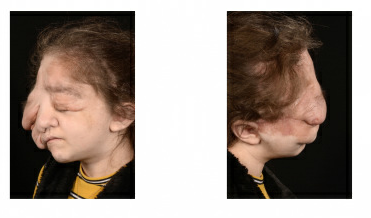

Supplement: Supplementary file 3 — Supplementary Figure 4. (A, B, C, D, E) A 23-year-old male who presented with right sphenoid wing dysplasia with herniation into the right orbit. (F, G, H, I, J) Clinical photographs taken one year after right sphenoid wing repair with titanium mesh. [file mmc3.docx]

**Supplementary Figure 4. (A, B, C, D, E, F, G, H, I, J)**


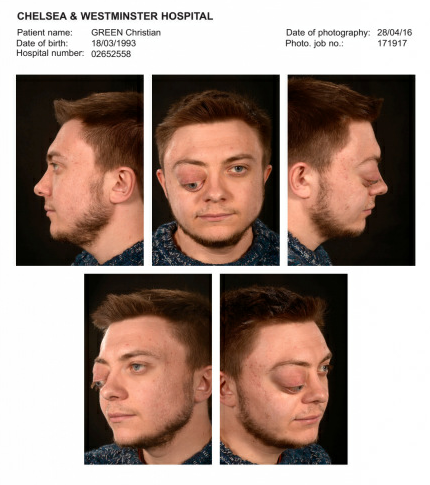


**EA**

**DA**

**CA**

**BA**

**AA**


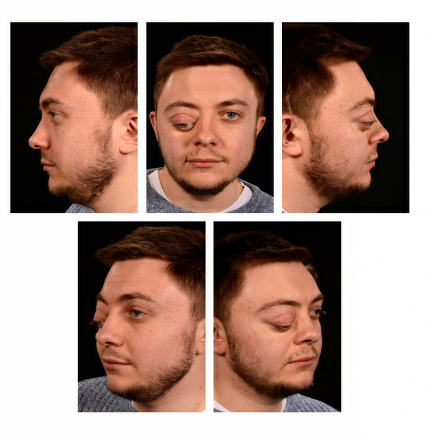


**FA**

**JA**

**IA**

**HA**

**G**

Supplement: Supplementary file 4 — Supplementary Figure 5. (A) Pre-operative 3D CT reconstruction demonstrating the radiological indications for surgery in one patient. (B, C, D) CT imaging demonstrating the radiological indications for surgery in one patient. [file mmc4.docx]

**Supplementary Figure 5. (A, B, C, D)**


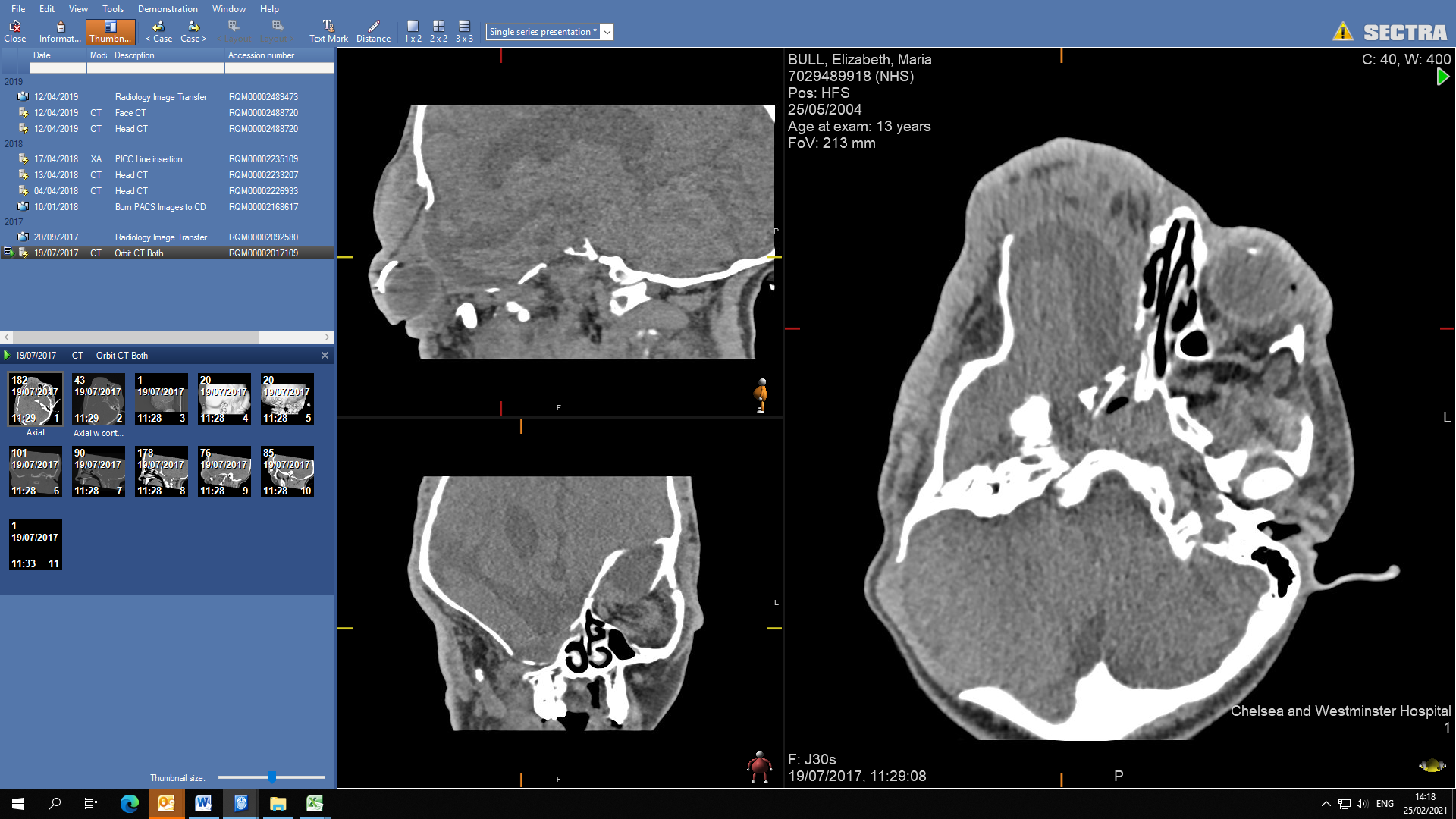

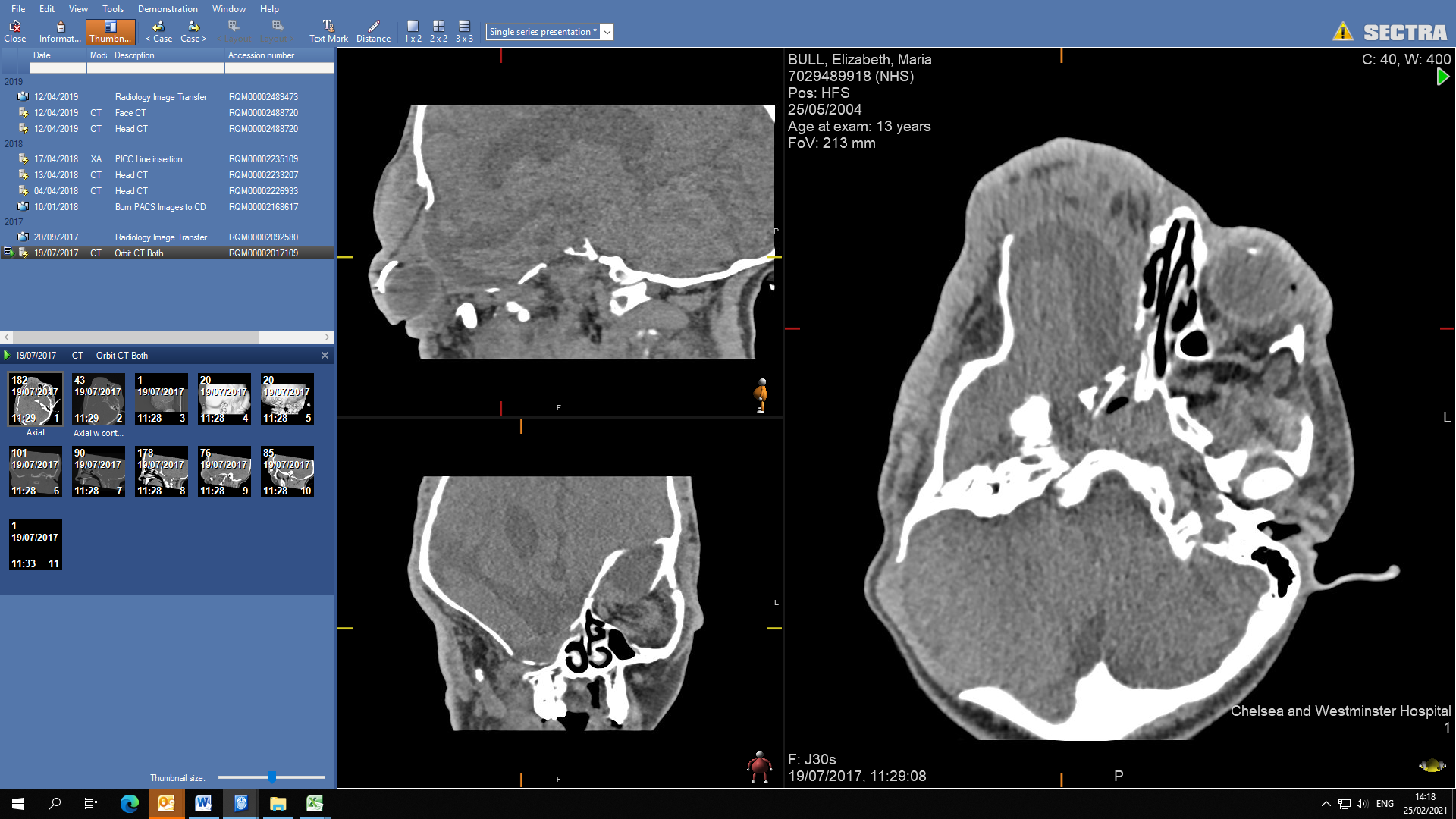


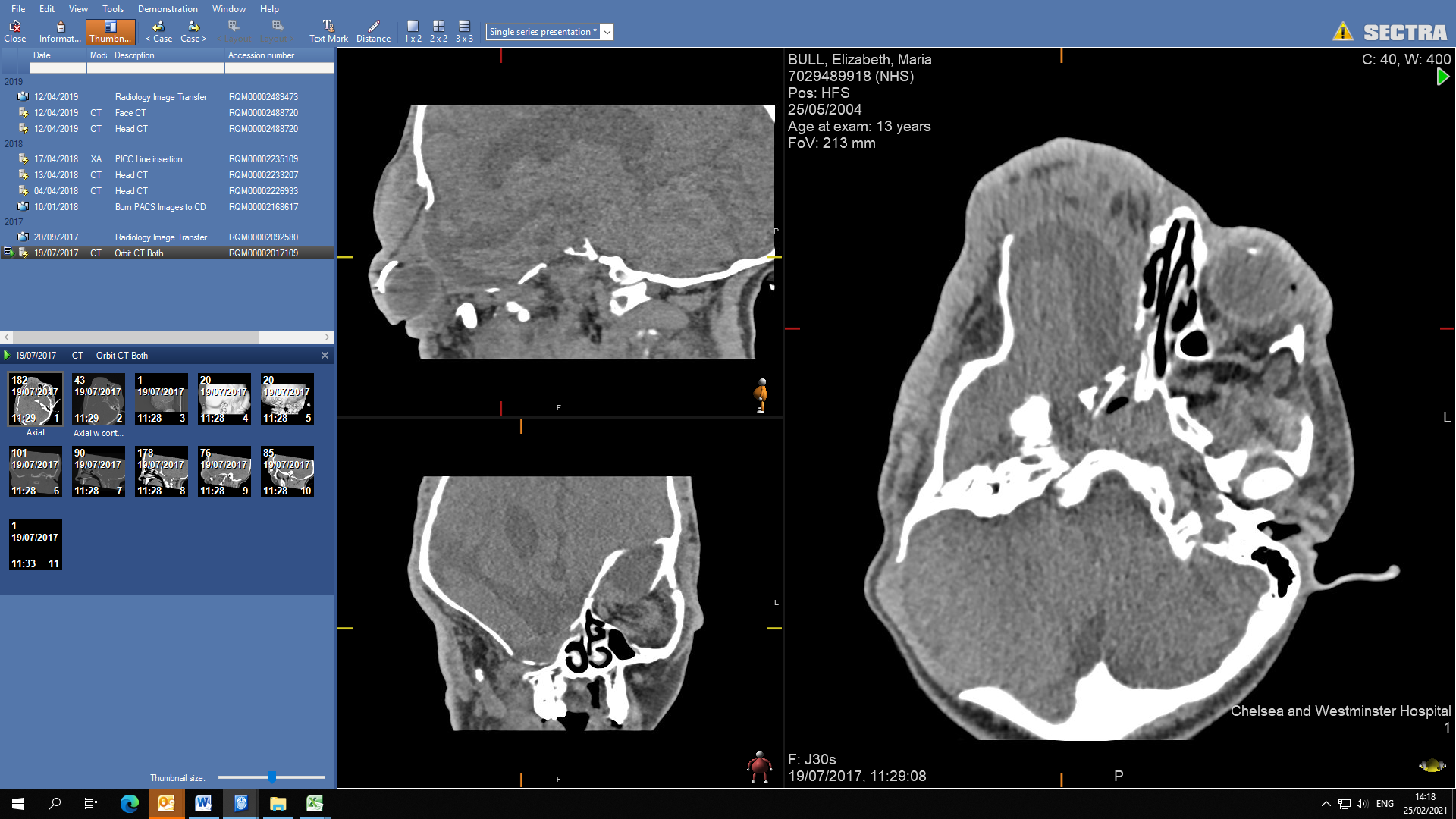


**D**

**CA**

**B**

**A**


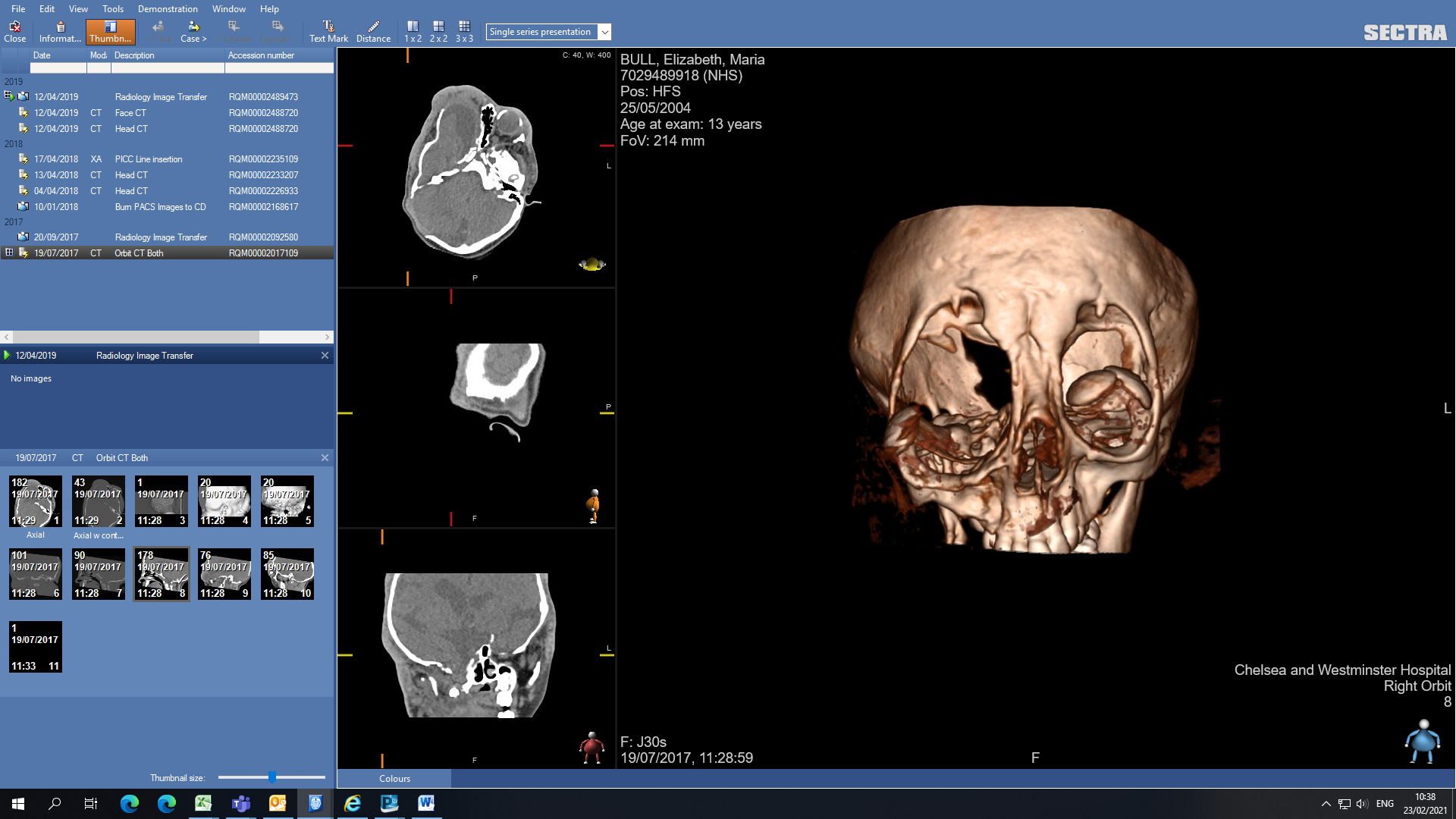

Supplement: Supplementary file 5 — Supplementary Figure 6. (A, B, C) A 2-year-old male patient that presented with left sphenoid wing dysplasia with herniation into the left orbit. Despite absent vision in the left eye at the time of surgery, surgical intervention was still indicated to arrest the progressive herniation demonstrated in consecutive pre-operative photographs of the patient aged 3 (D, E, F) and 5 years old (G, H, I).. [file mmc5.docx]

**Supplementary Figure 6. (A, B, C, D, E, F, G, H, I)**

**
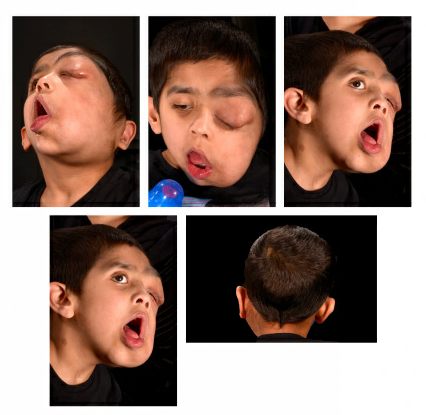

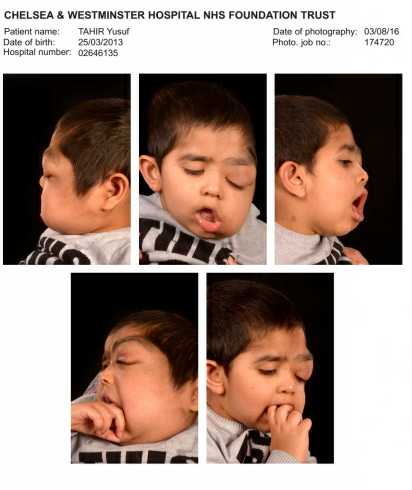

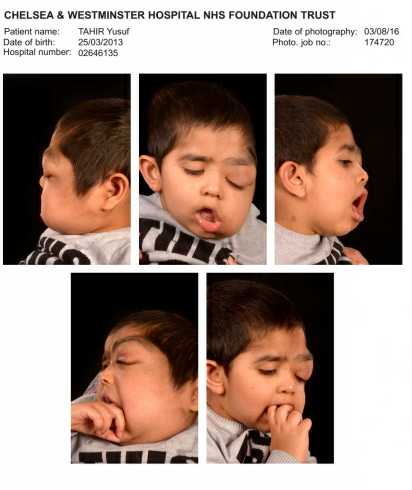

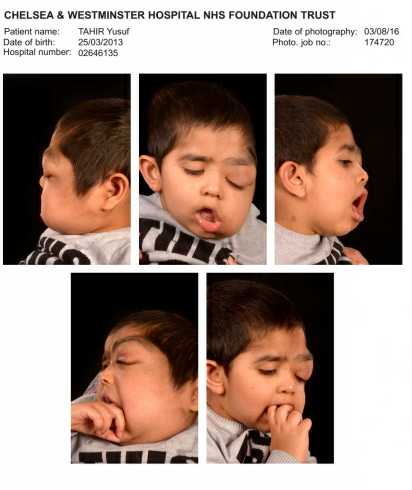

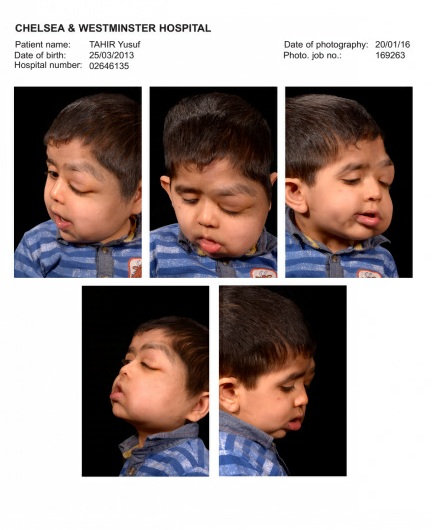
**

**IA**

**HA**

**GA**

**FA**

**EA**

**DA**

**CA**

**BA**

**AA**

Supplement: Supplementary file 6 [file mmc6.docx]
