# Supplementary material for: Reconstruction of Sphenoid Wing Dysplasia in Neurofibromatosis Type-1 Patients: An Evolving Technique
Source: JPRAS Open. 2021 Nov 10;31:67–71. doi: 10.1016/j.jpra.2021.10.002 (PMC8666329; doi:10.1016/j.jpra.2021.10.002)
Supplement: Supplementary file 2 — Supplementary Figure 3. (A, B, C) A 13-year-old female that presented with bilateral sphenoid wing dysplasia and herniation into both orbits. (D, E, F) Clinical photographs of the same patient 10 months later, highlighting the rapid advancing herniation into the right orbit. (G, H, I) Clinical photographs taken after bilateral sphenoid wing dysplasia repair using endoscopic decompression and a custom-made 3D titanium implant. [file mmc2.docx]

| **Age at surgery** | **Gender** | **Pathological Side** | **Co-morbidities** | **Implant** | **Decompression** | **Complications** |
| --- | --- | --- | --- | --- | --- | --- |
| 13 | Female | Right | Growth hormone deficiency | 3D titanium | Endoscopic | Sub-galeal CSF leak managed with a shunt |
| 14 | Female | Left | Growth hormone deficiency | 3D titanium | Endoscopic | Nil |
| 6 | Male | Left | Epilepsy  Learning difficulties | 3D titanium | Endoscopic | Nil |
| 24 | Male | Right | Nil | Titanium mesh | Milked CSF | Nil |
| 44 | Female | Left | Nil | Titanium mesh | Nil | Nil |

**Supplementary Figure 2.**
